# Safety assessment of Maillard reaction products of chicken bone hydrolysate using Sprague-Dawley rats

**DOI:** 10.3402/fnr.v60.27827

**Published:** 2016-03-23

**Authors:** Jin-Zhi Wang, Hong-Mei Sun, Chun-Hui Zhang, Li Hu, Xia Li, Xiao-Wei Wu

**Affiliations:** Institute of Food Science and Technology, Chinese Academy of Agricultural Sciences, Beijing, China

**Keywords:** Maillard reaction products, chicken bone hydrolysate, safety, rat, toxicology

## Abstract

**Background:**

The Maillard reaction products of chicken bone hydrolysate (MRPB) containing 38% protein, which is a derived product from chicken bone, is usually used as a flavor enhancer or food ingredient. In the face of a paucity of reported data regarding the safety profile of controversial Maillard reaction products, the potential health effects of MRPB were evaluated in a subchronic rodent feeding study.

**Methods:**

Sprague–Dawley rats (SD, 5/sex/group) were administered diets containing 9, 3, 1, or 0% of MRPB derived from chicken bone for 13 weeks.

**Results:**

During the 13-week treatment period, no mortality occurred, and no remarkable changes in general condition and behavior were observed. The consumption of MRPB did not have any effect on body weight or feed and water consumption. At the same time, there was no significant increase in the weights of the heart, liver, lung, kidney, spleen, small intestine, and thymus in groups for both sexes. Serological examination showed serum alanine aminotransferase in both sexes was decreased significantly, indicating liver cell protection. No treatment-related histopathological differences were observed between the control and test groups.

**Conclusion:**

Based on the results of this study, the addition of 9% MRPB in the diet had no adverse effect on both male and female SD rats during the 90-day observation. Those results would provide useful information on the safety of a meaty flavor enhancer from bone residue as a byproduct of meat industry.

The Maillard reaction is a well-known non-enzymatic interaction between reducing sugar and amino acid, peptide, or protein that can contribute markedly to the aroma, taste, and color of products (1). Maillard reaction products (MRP) have received particular attention because of their wide array of properties. They are well known for having aromatic, textural, and sensory properties (2–4), together with antioxidant abilities (5, 6). Since the aroma generation by Maillard pathways was reported in 1914, the food industry has patented flavor formations using it (2). For example, the Maillard reaction was used to increase the meaty aromas of enzyme-hydrolyzed wheat protein (7), and chicken bone extracts (CBEs) showed a golden brown color with increased amounts of pyrazine and sulfur compounds that improved the quality of flavor (8, 9). It is reported that thermally processed foods rich in MRP could have a beneficial impact on *ex vivo* low-density lipoproteins (LDL) oxidation (10). Some studies reported that the antioxidant activities of MRP, derived from peptide/protein/sugar systems, were considerably increased when protein was heated in the presence of reducing sugar (11, 12). A study showed that the protection of Caco-2 cells from free radical-, ferrous-, and cupric-induced cytotoxicity by casein was not altered by the Maillard reaction (10), and the MRP of casein showed no toxicity to Caco-2 cell at both low and high concentrations (10). It is also elaborated that the consumption of MRP is associated with certain positive biological actions, such as antioxidant and chemopreventive activities (13), and the indigestible melanoidins produced from the Maillard reaction displayed *in vivo* antioxidant, antimicrobial, and prebiotic activities in the small intestine that were beneficial to human health (14). MRP have been reported to have antioxidant activity in both the chemical model and food system (15).

On the other hand, MRP are considered to have some toxic substances, which have been reported to decrease the nutritional value of foods (16), in addition to diverse negative consequences such as protein damage (17) and decreased mineral availability (18). Besides, the Maillard reaction has been suggested to play an important role in the formation of acrylamide (19) and other kinds of heterocyclic amines (HAs) (20). HAs are an important group of food mutagens and potential carcinogens in rodents and primates (20). Many HAs are formed via the Maillard reaction from creatine, free amino acids, and monosaccharides, and the discovery of the adventitious formation of the potential cancer-causing HAs, especially in protein-rich foods of animal origin, has raised worldwide concerns (19, 20).

During the 2009 World Summit on Food Security, it was recognized that, by 2050, food production must increase by about 70% to feed the anticipated world population of nine billion people (21). It means an additional annual consumption of nearly 200 million metric tons of meat or animal source protein. Consequently, by-products, also named as non-conventional food sources, will be increased dramatically. For example, approximately 5.6–14 million tons and 16.6–41.5 million tons of chicken bone (CB) was produced in China and the whole world in 2012, respectively (22), which were mainly used as feed or discarded. CB is a valuable source of proteins and other nutrients, including approximately 51% moisture, 19% protein (35%–40% collagen), 9% fat, and 15% ash, including various types of mineral elements, especially calcium, iron, and phosphorus (8, 23). Hence, an interesting alternative would be to transform CB residue into a flavor ingredient by the Maillard reaction, which can generate flavor and color in the thermal processing of protein-rich foods (19). As a newly emerging natural flavor seasoning enhancer product, MRP derived from CB through Maillard reactions shows good flavor quality (3). Since it is derived from CB, the most valuable, abundant, and readily available byproduct, Maillard reaction products of chicken bone hydrolysate (MRPB) is recognized as a non-conventional food source, and is highly popular in Asia and Australia. At the same time, most of the studies on the effects of MRP are based on the results of *in vitro* tests, and MRPB has been questioned for its potential toxicity due to the possible formation of HAs and some unknown compounds.

Therefore, MRPB needed to be evaluated for its safety, as there is no well-documented history on its use as human food. A 13-week safety assessment on MRPB in Sprague-Dawley (SD) rats was thus performed in the present study to provide useful information on MRPB as a potential flavor enhancer and nutrient resource derived from CB.

## Materials and methods

### Test material

#### Preparation of chicken bone extracts

CBEs were prepared by hot-pressure extraction as reported previously (8). Briefly, CB residues were soaked in water at room temperature for 10 min to wash out the residual blood, and were then placed into a crane cage, hanging in hot-pressure extraction pots. They were then mixed with equal amounts of distilled water (w/v) and extracted at 135±0.5°C for 120 min. The resulting soup was filtered through a 200-mesh sieve to remove the bone residues, and the filtrate was poured into a standing pot, incubated at 85±1.0°C for 120 min. The supernatant (bone oil) was removed, while the aqueous layer was collected and concentrated by the vacuum condenser under the negative pressure of 0.1 MPa until the content of total solid reached about 40%. The concentrated CBE was stored at −20°C for further use.

#### Preparation of MRPB

Concentrated CBE was used as a substrate for hydrolysis. Fifty grams of CBE were added into 150 ml of water for an appropriate substrate concentration. Food-grade Protamex from Novozymes Co. Ltd (Beijing, China) was added to the slurry at a ratio of 0.5% (w/w) and hydrolyzed in a water-bathed vibrator under 40±1°C and pH 6.8±0.2 for 2 h. Subsequently, flavourzyme was supplemented and the slurry was hydrolyzed for another 2 h under the same conditions. Finally, the reaction was inactivated by heating at 95°C for 10 min.

To provide the reducing sugar and amino acid necessary for the Maillard reaction, system cysteine (0.5%, w/w), xylose (0.5%, w/w), and thiamine (0.5%, w/w) were added to 200 mL of hydrolyzed CBE. The slurry was subjected to the Maillard reaction in a high-pressure stainless reactor (Shanghai, China) at 105°C, pH 7.0. Samples (MRPB) were collected at 90 min of reaction and cooled down by a mixture of ice and water, and stored at −20°C prior to further analysis.

The fat, moisture, and ash contents of the MRPB were determined according to official methods of Association of Official Analytical Chemists (AOAC) (24). Crude protein was determined by the Kjeldahl method. Mineral analyses of the MRPB were carried out following a method with minor modifications (25). Briefly, 1.0 g of MRPB was digested with 8 ml of nitric acid (65%, v/v) and 2 mL of hydrogen peroxide (40%, v/v) on a focused microwave system using borosilicate vessels. Mineral analyses were carried out using inductively coupled plasma/atomic emission spectrophotometry (ICP/AES) instrument (SPS8000, KCHG, China). Sugars were determined by a phenol-sulfuric acid method using glucose as a standard for the method of AOAC (24). Peptides content, also named soluble hydrolyzed protein, was assessed by the Biuret's method, as described (3). Glutathione (GSH) was used as a standard and polypeptide content was described as the equivalent of GSH.

#### Preparation of the rodent diet

The experimental sterilized rat diet used in the study was prepared in meal form by Vital feed company (Beijing, China), based on the rodent diet standard (26). MRPB was added to the basic rodent diet to a final content of 0, 1, 3 and 9% (w/w), respectively. All ingredients were ground to a similar particle size to ensure a homogeneous mixture. All diets were analyzed quantitatively for nutrient composition. The fat, moisture, ash, protein and minerals content were determined as that of MRPB described before. Total amino acid composition was analyzed as described (3). The amino acid compositions for the samples were measured by L-8900 Amino Acids Automatic Analyzer (Hitachi LTD, Japan). Crude fiber was determined by the method of AOAC. Carbohydrate and energy content was calculated by the method of AOAC (24).

#### Laboratory animals and housing

Five-week old male and female SD rats were purchased from Charles River China (Beijing, China) and maintained in the animal facility of the Institute of Genetics and Developmental Biology, Chinese Academy of Sciences, according to the Institutional Rules for Animal Experimentation. The Animal Care and Use Committee for Institute of Agro-food Science and Technology, Chinese Academy of Agricultural Sciences approved the protocol of this experiment. Rats were housed five per cage, and given free access to drinking water and basal diet, under controlled conditions of humidity (60±10%), lighting (12 h light/dark cycle), and temperature (22±2°C). Rats were acclimated for 1 week prior to the start of the experiment and randomly assigned to four groups following computerized randomization based on body weight (*n*=5/sex/group). During the test period, rats were fed the prepared diets containing 0, 1, 3, and 9% MRPB, and they were grouped as control, low dose, middle dose, and high dose, respectively.

### Experimental protocol

During the experimental period, all animals were observed clinically twice daily. Body weight and food consumption were recorded weekly. On day 90, after 12 h of food withdrawal, all animals were weighed and then sacrificed after collection of blood samples from the abdominal aorta (27). ETDA-2K was used as the anticoagulant for the blood samples. The collected blood samples were processed for biochemistry and hematology, respectively. Referring to the reported method (28), we combined fecal samples of same-gender rats to test the differences between the experimental group and the control group.

### Blood biochemistry

The following serum biochemical parameters were measured: urea (BUN), alanine aminotransferase (ALAT), cholesterol, total protein, albumin (ALB), triglyceride (TG), creatinine (CREA), glucose, calcium, magnesium, and phosphorus. All analyses on blood serum were performed with the Hitachi automatic biochemical analyzer, 7020 (Hitachi, Japan), using the relevant kits for each parameter.

### Hematology

Hematology characteristics were assessed using a Vet ABC, Animal Blood Counter (Analysis instruments AB, Stockholm, Sweden) on the following parameters: White blood cells (WBC), red blood cells (RBC), hematocrit (HCT), hemoglobin (HGB), mean corpuscular hemoglobin (MCH), mean corpuscular volume (MCV), platelets (PLT), red cell distribution width (RDW), and mean corpuscular hemoglobin concentration (MCHC). The differential count was performed for neutrophils (NEU), lymphocytes (LYM), eosinophils (EOS), basophils (BAS), and monocytes (MON). The histologic and diagnostic work reported herein was undertaken between December 23, 2013, and February 25, 2013, in compliance with the Good Laboratory Practices (GLP) requirements specified in the Organization for Economic Co-operation and Development (OECD) ‘Principles of Good Laboratory Practices’ (29).

### Organ weights, gross necropsy, and histopathology

A thorough necropsy was performed and the following organs were excised and weighed: adrenals, brains, heart, kidneys, liver, lung, ovaries, small intestine, spleen, testes, epididymides, thymus, and uterus. Paired organs (adrenals, epididymides, kidneys, ovaries, and testes) were weighed as a total of right and left. Sections from the above organs were fixed in 10% buffered formaldehyde for histological processing. Tissue samples were embedded in paraffin sections, 3–5 µm thick, and were then stained with standard hematoxylin and eosin for light microscopy. Microscopy observations were performed with a Vanox-S microscope (Olympus, Japan). Histopathological assessment was first performed on all tissues of the control and high-dose group, and for the liver and kidneys in all groups. If a chemical treatment-related change appeared at the highest dose, the relevant tissue(s) from the lower dose groups were then also examined.

### Statistical analysis

Compositional data are presented as means. Data obtained from the animal studies were analyzed separately for each sex and presented as mean±SD, where appropriate. Dunnett's multiple comparison (*P*<0.05) was applied. If significant heterogeneity of variance was detected, the Student's t-test for comparing treatment and control groups was employed. All statistical analyses were carried out using SAS release 9.1 (30).

## Results and discussion

### Characterization of MRPB and experimental diets

The composition of MRPB and rodent diet is presented in Tables 1 and 2, respectively. Concentrations of proximate analyses, fiber, amino acids, vitamins, and minerals (calcium and phosphorus) were similar to those reported for PMI_Nutrition International, LLC Certified Rodent LabDiet 5002 (31), although some analytes fell outside this range. Such compositional differences were attributable to the addition of MRPB. Gross energy content ranged from 360 kcal/100 g (control) to 364 kcal/100 g, all well above 290 kcal/100 g typical diet for a weaning rat and 250 kcal/100 g for a mature rat (32). Because rats compensate for feed energy density with altered feed consumption to meet caloric needs (32), the high gross energies of these experimental diets were considered to have no nutritional impact.

**Table 1:** Compositional analysis of MRPB

Components	Content	Minerals	Concentration (mg/kg)
Dry matter (%)	54.12	P	1230.90
Ash (%)	7.18	Ca	218.00
Fat (%)	0.77	Fe	10.03
Protein (%)	37.63	Zn	2.88
Sugar (g/100 g)	2.98	K	2217.10
Peptide (mg/g)	472.70	Na	5711.20

Data is presented as mean values. MRPB, Maillard reaction products of chicken bone hydrolysate.

**Table 2:** Composition of rodent diet containing MRPB (9, 3, 1, and 0%)

	9%	3%	1%	Control
Proximates				
Moisture (g/100 g)	8.395	8.625	8.57	8.27
Ash (g/100 g)	7.14	6.835	6.66	6.63
Protein (g/100 g)	28.155	23.545	22.185	21.21
Fat (g/100 g)	3.94	3.61	4.26	3.77
Fiber (g/100 g)	3.16	3.14	3.39	3.43
Carbohydrate (g/100 g)	52.37	57.385	58.325	60.13
Energy (Kcal/100 g)	362.02	360.75	364.79	363.81
Amino acids(mg/g)				
Asp	15.91	14.20	13.39	12.43
Thr	6.73	6.12	5.71	5.32
Ser	8.75	8.26	7.95	7.11
Glu	39.61	36.72	35.09	32.39
Gly	15.13	10.22	7.93	6.52
Ala	10.94	9.21	7.73	7.21
Cys	2.46	3.22	2.69	2.72
Val	7.46	7.49	6.86	6.71
Met	2.64	2.66	2.31	1.79
Ile	6.24	5.74	5.49	5.21
Leu	12.98	12.29	11.45	11.11
Tyr	4.00	3.80	3.60	3.54
Phe	8.31	7.63	7.33	6.94
Lys	9.31	7.90	7.11	6.99
His	4.12	3.83	3.57	3.36
Arg	11.61	9.92	9.18	8.20
Pro	14.61	12.22	10.32	9.91
Total amino acids(mg/g)	180.81	161.42	147.71	137.46
Minerals				
Calcium (g/100 g)	1.03	1.12	1.29	1.21
Phosphorus (g/100 g)	0.79	0.98	0.315	0.65

Crude protein concentrations in the high dose of MRPB (9%) diet exceeded LabDiet 5002 typical values. Proteins are a necessary component of the diet of humans and other mammals (33). Because of the sensitivity of dietary proteins to digestion and the minimal potential for absorption of intact proteins from the GI system (34), proteins can be degraded into constituent amino acids that are efficiently absorbed. Therefore, the overwhelming majority of dietary proteins possess no potential for systemic toxicity (35). Accordingly, the consumption of protein is not normally associated with adverse effects (36), though in toxicology, it is well accepted that dose influences toxicological outcome (37).

The determination of amino acid requirements for adult rats is difficult because of the flat dose-response curves that occur for many amino acids (38). Amino acids in different groups ranged from 137.46 (control) to 180.81 mg/g (high dose of MRPB), which is not uncommon, because the requirement for an amino acid tends to increase with dietary protein (32). Besides, if the diet contains a mixture of proteins, both the content and bioavailability of the amino acids in the different proteins must be considered for maximum growth (32). Our previous study demonstrated that the CBEs used for MRPB in this study shows an ideal protein digestibility-corrected amino acid score for adult *in vitro* (8), and indicated that flavor amino acids accounted for 72%~ 74% of total free amino acids in MRPB with good taste acceptability (3). During the experiment, the obvious expectancy on the diet containing MRPB from those tested rats were observed (data not shown).

The concentrations of all analytes and/or their dietary equivalents satisfied the NRC recommended minimum dietary intakes of these nutrients for maintenance of the adult laboratory rat, and were well below the dietary concentrations reported (if available) to cause adverse health effects (32). Heavy metals (LOQ for arsenic=0.50 ppm; LOQ for lead=1.00 ppm; LOQ for mercury=0.100 ppm; LOQ for cadmium=0.250 ppm) were not detected in any diet (data not shown).

### Body weight and food consumption

**Fig. 1:**
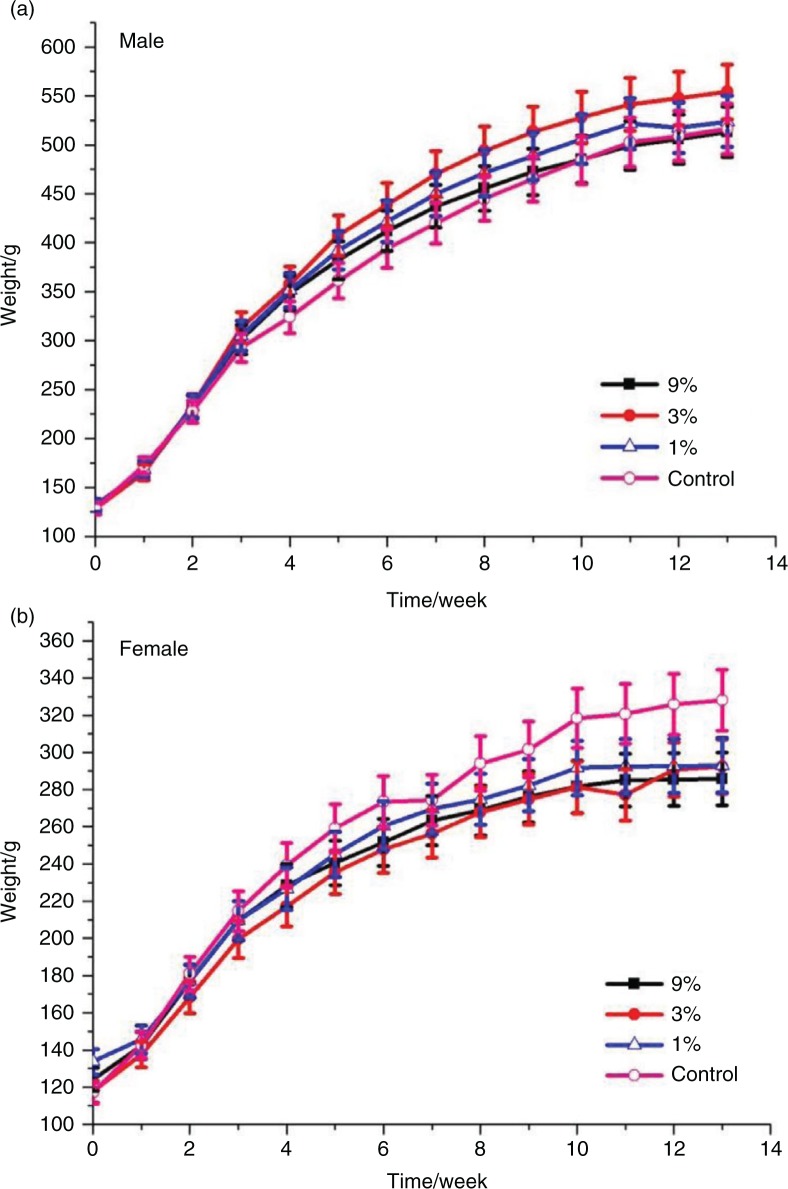
Growth curves for male and female Sprague-Dawley rats treated with MRPB for 13 weeks. (a) Cumulative bodyweight curves for males rats during 13-week study; (b) Cumulative bodyweight curves for female rats during 13-week study. MRPB, Maillard reaction products of chicken bone hydrolysate; 9, 3, and 1%, and control represents 9, 3, 1, and 0% of MRPB in diet for rats.

**Fig. 2:**
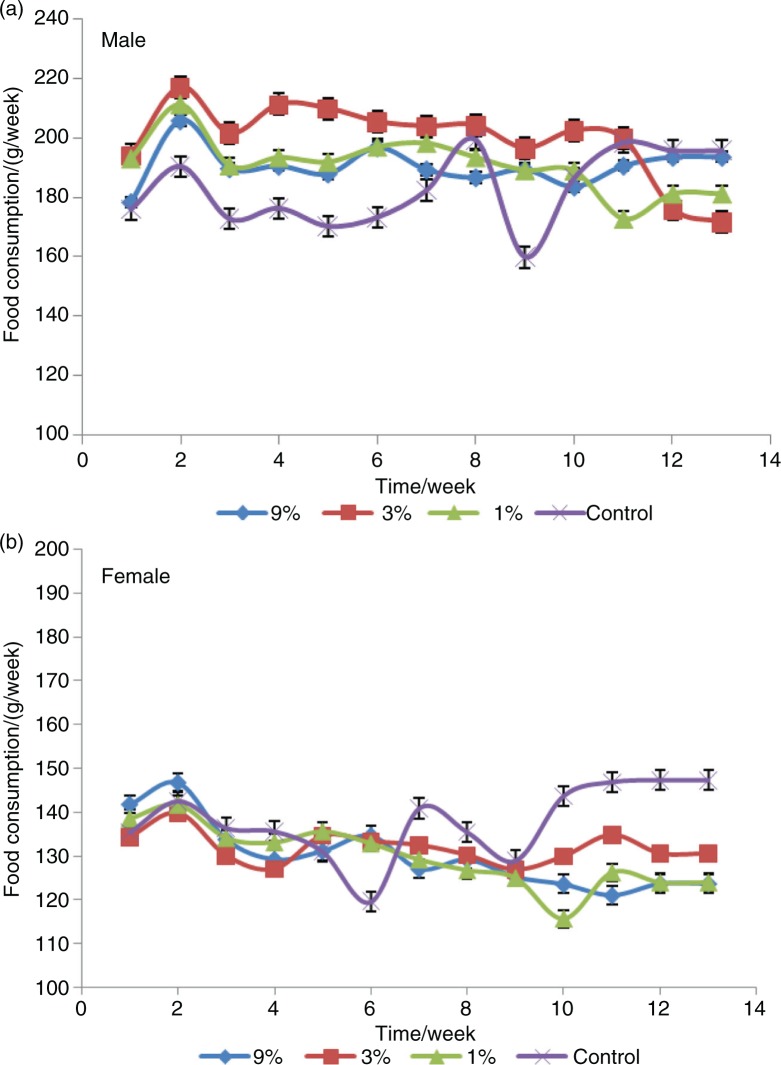
Food consumption for male and female Sprague-Dawley rats treated with MRPB for 13 weeks. (a, b) represents food consumption for male and female SD rats during the 13-week study, respectively. MRPB, Maillard reaction products of chicken bone hydrolysate; 9, 3, and 1%, and control represents 9, 3, 1, and 0% of MRPB in diet for rats.

**Fig. 3:**
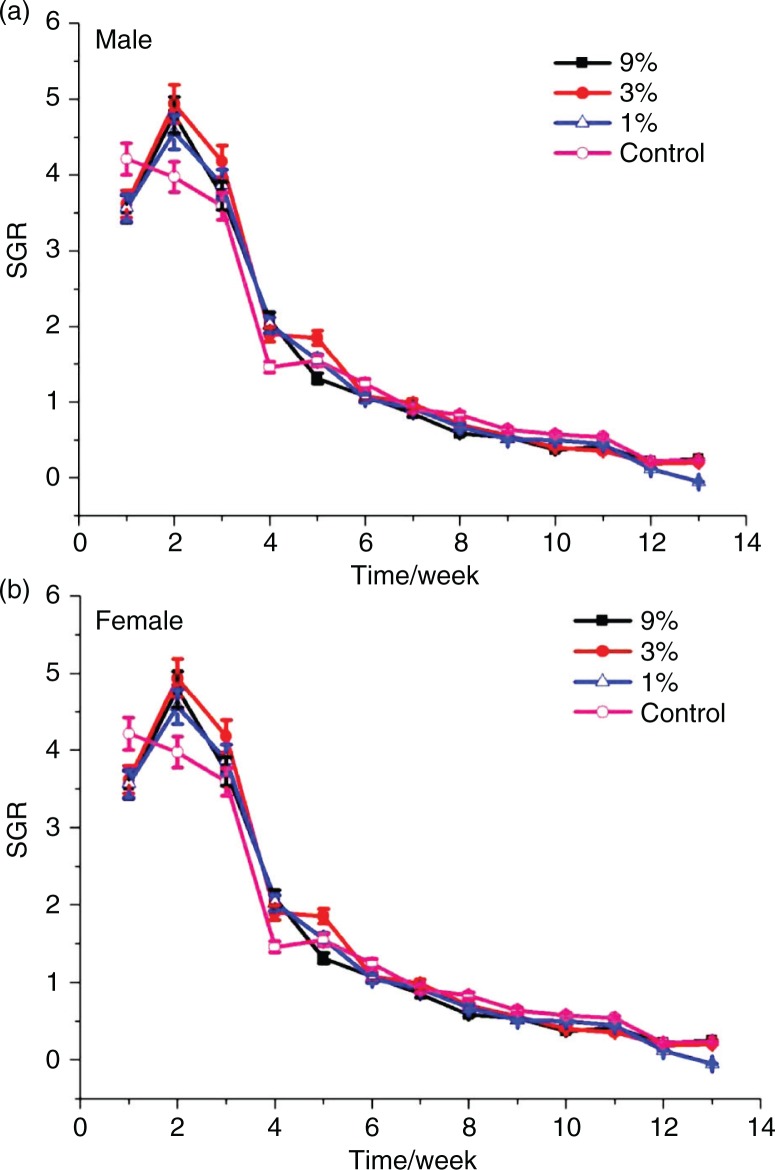
Specific growth rates (SGR) for male and female Sprague-Dawley rats treated with MRPB for 13 weeks. (a) SGR for male SD rats during 13 weeks; (b) SGR for female SD rats during the 13-week study. The SGR for weight was calculated as SGR (%)=(lnw2-lnw1)×100/feeding days, where w1 and w2 are start and final weights, respectively, and ln is the natural logarithm. MRPB, Maillard reaction products of chicken bone hydrolysate; 9, 3, and 1%, and control represents 9, 3, 1, and 0% of MRPB in diet for rats.

The animals were observed twice daily for well-being. Body weights and food consumption were measured weekly, and specific growth rates were calculated. During the 13-week experiment, no treatment-related signs of adverse effects in clinical appearance on the animals were observed. There were no treatment-related differences in body weight gain of rats of either sex (Fig. 1). The body weights of male rats fed with MRPB were higher than that of control group, whereas female rats fed the diet containing MRPB tended to have lower body weights compared with those of the control group, but there were no statistically differences between the sexes for both treatment and control (*P*>0.05). Therefore, the differences in mean male body weights were considered non-adverse, and this result is similar with reported findings (39). As shown in Fig. 2, at week six, food consumption decreased in the male vehicle, and it was less than the three treatment groups (*P*<0.05), while that of female vehicle decreased at 6 and 9 weeks. Similarly, Delaney et al. observed the significant difference (*p*<0.05) in food consumption between the male and control groups during one feeding interval (days 84–91), even though other food consumption was similar (39). Interestingly, after 10 weeks of treatment, the food consumption in the female vehicle group was significantly higher than that of three groups fed with MRPB (*P*<0.05). Diets with MRPB showed no effect on specific growth rate and feed efficiency ratio of SD rats (Fig. 3). There were also no significant differences in water consumption between the treatment and control groups throughout the study (data not shown).

### Serum biochemistry and hematology

Serum biochemical results on both sexes of rats are shown in Table 3. It can be seen that, by the end of 13-week treatment period, the blood glucose levels in SD rats fed with MRPB or the control group in the present study were within the normal range of values, which was 2.32–6.06 and 3.20–6.23 mmol/L for female and male SD rats, respectively (40). There were no treatment-related adverse effects in the mean values of clinical chemistry variables, except that activity of ALAT in rats consuming MRPB was lower than that of control group (*P*<0.05).

**Table 3:** Serum biochemistry of rats fed with feed containing MRPB (9, 3, 1, and 0%) for 13 weeks

	Male				Female			
		
	9%	3%	1%	Control	9%	3%	1%	Control
Glucose (mmol/L)	5.22±0.68	5.57±0.79	5.92±0.45	5.79±0.79	5.03±0.40	7.62±4.69	5.15±0.49	5.31±0.41
Total protein (g/L)	71.86±4.26	70.02±5.92	71.94±4.67	66.72±4.41	77.80±5.44	71.92±6.08	72.02±4.91	77.38±6.03
Albumin (g/L)	33.48±0.96	34.4±2.72	33.52±2.02	32.98±1.78	40.42±3.53	37.44±3.78	37.72±2.20	40.70±2.99
Cholesterol (mmol/L)	1.99±0.27	1.93±0.61	2.05±0.54	1.80±0.33	2.37±0.45	2.18±0.38	2.03±0.47	1.83±0.31
Triglyceride (mmol/L)	0.54±0.23	0.52±0.18	0.56±0.26	0.60±0.23	0.48±0.08	0.74±0.47	0.46±0.11	0.56±0.09
Calcium (mmol/L)	2.65±0.20	2.69±0.24	2.80±0.31	2.93±0.60	2.70±0.10^B^	2.72±0.24^B^	3.24±0.09^A^	2.54±0.10^B^
Magnesium (mmol/L)	1.06±0.05	1.15±0.1	1.05±0.06	1.04±0.11	1.12±0.02	1.17±0.22	1.07±0.07	1.10±0.15
Phosphorus (mmol/L)	2.33±0.22	2.43±0.19	2.35±0.20	2.62±0.91	2.31±0.14	2.61±1.39	2.42±0.21	1.44±1.00
Urea (mmol/L)	5.54±0.53	6.12±1.00	6.41±1.39	6.77±0.71	7.18±1.62	6.37±1.43	6.00±0.65	6.51±1.55
Creatinine (µmol/L)	23.80±2.05^b^	25.40±2.88^ab^	27.20±2.59^a^	24.00±2.00^b^	26.40±3.13	31.40±3.91	30.20±1.92	27.40±5.13
ALAT (U/L)	49.20±7.91^b^	51.00±6.60^b^	55.40±14.82^ab^	67.80±11.76^a^	68.8±19.66^B^	48±5.89^B^	40.6±8.20^B^	69.40±27.32^A^

MRPB, Maillard reaction products of chicken bone hydrolysate; 9, 3, and 1%, and control represents 9, 3, 1, and 0% of MRPB in diet for rats; ALAT, alanine aminotransferase; ALAT, alanine aminotransferase.

The number of animals was five rats/sex/group; data are presented as group mean values±SD. Lower case superscript letters indicate significant differences among male rats, while upper case superscript letters indicate significant differences among female rats (*p*<0.05).

Table 4 shows the total and differential count of WBC, RBC, HCT, HGB, MCH, MCV, PLT, RDW, MCHC, NEU, LYM, EOS, BAS, and MON values obtained in different groups of rats. Hematology analysis revealed that there were no significant differences observed in female rats. Several statistically significant differences were observed between the four groups of male rats. The MCHC of male rats was slightly higher in the 3%-dose treatment group fed with MRPB, whereas the MCH of this group was slightly lower than that of the control group. There were no other treatment-related changes in hematology and serum biochemistry parameters in rats fed with the diet containing MRPB, in the present study.

**Table 4:** Hematological values of rats fed with feed containing MRPB (9, 3, 1, and 0%) for 13 weeks

	Male				Female			
		
	9%	3%	1%	Control	9%	3%	1%	Control
WBC (10^9^/L)	9.08±3.93	10.18±4.53	8.26±2.73	9.76±3.67	7.56±1.44	7.14±2.06	8.98±1.70	8.00±2.01
RBC (10^12^/L)	7.69±1.45	8.76±0.77	8.04±1.05	8.43±0.99	7.44±0.82	7.90±0.81	8.01±0.75	8.37±0.99
HGB(g/L)	175.4±27.18	197.8±19.15	183.4±21.65	192.6±14.54	179.8±18.53	193.2±19.21	184.6±14.19	190.8±19.29
HCT (%)	39.38±6.29	43.06±4.21	39.38±4.12	43.50±3.94	39.00±4.12	39.94±4.60	40.16±2.78	43.46±3.98
MCV (fL)	51.48±2.13	49.16±1.74	49.12±1.61	51.72±1.64	52.52±1.78	50.56±1.68	50.26±1.53	52.06±2.00
MCH (pg)	22.98±1.36	22.58±0.72	22.88±1.50	22.96±1.22	24.20±0.69	24.50±1.09	23.10±1.66	22.90±1.57
MCHC (g/l)	446±14.93^ab^	459.40±4.51^a^	454.5±15.02^ab^	443.4±12.5^b^	461.2±4.09^B^	484.4±12.8^A^	460±25.88^B^	439.2±15.12^B^
RDW(%)	13.34±0.05^ab^	12.98±0.46^b^	13.50±0.32^a^	13.08±0.44^ab^	13.50±0.30	13.70±0.31	13.34±0.93	13.38±0.9
PLT (10^9^/L)	1272.4±435.08	1060.6±237.77	1158.6±228.98	1313.6±245.24	1051.2±486.22	1138±381.33	1293.8±290	1013.8±179.06
Differential count								
LYM (%)	53.42±11.03^ab^	57.66±3.07^ab^	48.38±5.88^b^	62.56±14.94^a^	59.72±17.67	48.66±6.91	58.50±7.17	46.22±16.43
MON (%)	1.40±1.01	1.26±1.24	1.56±1.73	1.34±1.01	1.16±1.21^AB^	1.72±0.85^AB^	0.44±0.40^B^	3.42±3.56^A^
NEU (%)	40.98±9.42^ab^	38.02±3.43^ab^	46.08±9.04^b^	31.84±11.82^a^	36.44±16.23	43.84±4.96	35.40±8.56	43.04±6.21
EOS (%)	4.14±3.07	2.70±0.91	3.28±2.30	3.90±3.41	2.14±0.78	3.46±1.79	4.80±2.34	3.90±2.73
BAS (%)	0.00±0.00^b^	0.36±0.41^ab^	0.70±0.62^a^	0.36±0.35^ab^	0.54±0.21	1.52±1.90	0.86±0.42	3.42±5.55

MRPB, Maillard reaction products of chicken bone hydrolysate; 9, 3, and 1%, and control represents 9, 3, 1, and 0% of MRPB in diet for rats; ALAT, alanine aminotransferase. WBC, white blood cell count; RBC, red blood cell count; HGB, hemoglobin concentration; HCT, hematocrit; MCV, mean corpuscular volume; MCH, mean corpuscular hemoglobin; MCHC, mean corpuscular hemoglobin concentration; RDW%, red blood cells volume distribution width; PLT, platelets; LYM, lymphocytes; MON, monocytes; NEU, neutrophils; EOS, eosinophils; BAS, basophils.

The number of animals was five rats/sex/group; data are presented as group mean values±SD. Lower case superscript letters indicate significant differences among male rats, while upper case superscript letters indicate significant differences among female rats (*p*<0.05).

### Organ weights, gross necropsy, and histopathology

The absolute organ weights and organ-to-body weight ratios are presented in Table 5. The organs weighted include brain, heart, lung, spleen, kidneys, liver, small intestine, thymus, adrenals, testis, epididymis, uterus, and ovaries. Both of the absolute weight and organ-to-body weight ratios of male rats fed with diet containing MRPB (9, 3, and 1%) showed no difference from that of the control group except the kidney-to-body weight of medial-dose (3%) group. These results indicated MRPB did not affect most of the organs in SD rats, but the high protein contained in MRPB may impose a certain burden on the kidneys. Interestingly, the heart-to-body weight ratio, liver-to-body weight ratio, lung-to-body weight ratio of the medial-dose (3%) female group decreased significantly (*p*<0.05) compared with that of the control group. And the absolute weights of the heart, liver, lung, and kidneys of this group were observed to decrease significantly (*p*<0.05), too.

**Table 5:** Absolute and relative organ weights of rats fed with feed containing MRPB (9, 3, 1, and 0%) for 13 weeks

	Male				Female			
		
	9%	3%	1%	Control	9%	3%	1%	Control
Body weight(g)	505.9±9.13	547.69±55.09	525.64±50.43	509.39±59.96	285.32±19.7	290.77±15.87	292.69±13.18	325.96±26.28
Heart	1.81±0.19	2.08±0.36	1.82±0.40	1.71±0.23	1.15±0.16^A^	0.94±0.09^B^	1.21±0.13^A^	1.23±0.04^A^
Brains	2.04±0.07	–	–	1.85±0.18	1.92±0.09	–	–	1.85±0.09
Liver	12.64±0.85	15.33±2.92	13.66±2.34	12.95±2.19	8.58±0.85^A^	7.12±0.60^B^	7.08±0.6^B^	8.70±0.68^A^
Lung	1.90±0.28	1.96±0.30	2.26±0.52	1.97±0.26	1.52±0.16^AB^	1.36±0.04^B^	1.45±0.11^AB^	1.72±0.39^A^
Kidneys	3.48±0.18^ab^	3.72±0.65^a^	3.33±0.54^ab^	3.09±0.28^b^	2.25±0.14^A^	1.82±0.18^B^	2.00±0.11^B^	2.02±0.23^B^
Spleen	0.82±0.10	0.81±0.17	0.85±0.13	0.8±0.19	0.58±0.16	0.46±0.04	0.52±0.09	0.56±0.05
Small intestine	8.41±1.03	7.94±2.31	8.43±1.34	8.84±1.22	6.54±1.09^AB^	5.76±0.55^B^	5.92±0.37^B^	8.40±3.30^A^
Thymus	0.52±0.09	0.44±0.04	0.42±0.13	0.44±0.08	0.35±0.06	0.31±0.04	0.39±0.10	0.34±0.05
Adrenals	0.05±0.01	–	–	0.07±0.02	0.08±0.02	–	–	0.13±0.07
Uterus		–	–	–	0.70±0.10	–	–	0.37±0.14
Testes	3.38±0.39	–	–	3.3±0.22		–	–	–
Epididymides	0.66±0.05	–	–	0.54±0.12		–	–	–
Ovaries		–	–	–	0.16±0.02	–	–	0.27±0.08
Relative weight								
Heart	0.36±0.04	0.38±0.04	0.35±0.09	0.34±0.04	0.40±0.04^A^	0.32±0.03^B^	0.41±0.05^A^	0.38±0.04^A^
Brains	0.40±0.01	–	–	0.36±0.01	0.67±0.04	–	–	0.57±0.04
Liver	2.50±0.15	2.80±0.25	2.60±0.30	2.54±0.15	3.00±0.26^A^	2.44±0.12^B^	2.43±0.27^B^	3.00±0.26^A^
Lung	0.38±0.06	0.36±0.04	0.43±0.11	0.39±0.06	0.53±0.06^A^	0.47±0.03^B^	0.50±0.03^B^	0.53±0.06^A^
Kidneys	0.69±0.03	0.68±0.07	0.63±0.09	0.61±0.03	0.79±0.06^A^	0.63±0.09^B^	0.68±0.05^B^	0.79±0.06^A^
Spleen	0.16±0.02	0.15±0.02	0.16±0.02	0.16±0.02	0.20±0.05	0.16±0.01	0.18±0.03	0.20±0.05
Small intestine	1.66±0.19	1.45±0.28	1.60±0.30	1.74±0.19	2.29±0.36	1.98±0.15	2.03±0.20	2.29±0.36
Thymus	0.10±0.02	0.08±0.01	0.08±0.03	0.09±0.02	0.12±0.01	0.11±0.02	0.13±0.03	0.11±0.01
Adrenal gland	0.01±0.00	–	–	0.01±0.00	0.03±0.01	–	–	0.03±0.01
Uterus	–	–	–	–	0.24±0.04	–	–	0.22±0.04
Testes	0.67±0.09	–	–	0.67±0.09	–	–	–	–
Epididymis	0.13±0.01	–	–	0.11±0.01	–	–	–	–
Ovaries	–	–	–	–	0.05±0.01	–	–	0.08±0.01

MRPB, Maillard reaction products of chicken bone hydrolysate; 9, 3, and 1%, and control represents 9, 3, 1, and 0% of MRPB in diet for rats; ALAT, alanine aminotransferase. The number of animals was five rats/sex/group; data are presented as group mean values±SD. Relative organ weights expressed as g/100 g body weight. Small intestinal length and relative length are expressed as cm and cm/g body weight. Lower case superscript letters indicated significant differences among male rats, while upper case superscript letters indicate significantly differences among female rats (*p*<0.05).

Histopathological observations were conducted for the organs and tissues of all scheduled necropsied rats in the control and high dose (9% of MRPB) groups at the end of 13 weeks. During the necropsy, there were no gross pathological findings. Results of the histopathological examinations did not reveal any changes in the intestinal tract or related organs. Therefore, no pathologically relevance was found to explain the identified differences in organ weights of the medial-dose (3%) female group in the current study.

## Discussion

To evaluate the safety of MRPB used as flavor enhancer or food ingredient, the current 13-week subchronic toxicity study was performed on SD rats fed with diets containing different amounts of MRPB (9, 3, and 1%). Results showed that the growth rate of the rats fed with the diet containing MRPB did not differ significantly from that of the control group, indicating that the selected amount of MRPB did not affect the growth of SD rats.

MRP from potato chips and French fries were reported to have acrylamide, and it has been classified as a probable human carcinogen (41) and is an effective clastogen formed by a Maillard reaction when food rich in protein and carbohydrates undergo thermal processing at temperatures of 120°C or higher (42, 43). The presence of acrylamide in a range of fried and oven-cooked foods, which had the typical Maillard reaction during processing, have caused worldwide concern because this compound has been classified as probably carcinogenic in humans (19). The harmful effect of acrylamide is mainly displayed in the inhibition of weight gain (43). However, there was no significant difference on body weight for both male and female rats fed with diet containing 9% of MRPB in our study. Interestingly, the body weights of the male rats increased slightly but decreased in female rats fed with MRPB, comparing with that of the control group, indicating that MRP from CB did not have a harmful effect related to acrylamide, at least not in male rats. There was no evidence of anemia in SD rats fed with MRPB. The lack of a decrease in TP and ALB suggested that MRPB had no detrimental effects on both male and female rats. Similarly, an *in vitro* study also demonstrated MRP produced in a casein-sugar model having no toxicity to Caco-2 cell at both low and high concentrations (0.5 and 2 mg/ml) (6). Besides, it was also reported that the increased intake of the MRP reduced phosphorous digestibility in male adolescents (44), but the serum phosphorous in both male and female SD rats fed different amounts of MRPB showed no significant difference in this study (*P*>0.05, Table 3), indicating that MRPB had no effect on phosphorous metabolism in SD rats during the 90-day observation.

Significant alterations in mean corpuscular hemoglobin concentration (MCHC), lymphocytes (LYM), neutrophils (NEU), basophils (BAS), and monocytes (MON) were sporadically observed in rats of both genders fed with MRPB, and all the values were within the normal range of historical records of SD rats (40). Therefore, these were interpreted as a non-treatment-related difference, which means that MRPB (1–9%) in diet of SD rats did not affect the performance of those cells. No significant differences were observed in most of serum biochemistry findings, except that of ALAT of both sex and Ca of female rats given low-dose of MRPB (1%). ALAT is considered as a signature of liver cell protection (45). Therefore, the slight decrease of ALAT in the medium- and high-dose male groups and all female groups fed with MRPB indicated that MRPB may have a potential protection effect on rats’ liver. Similarly, it was previously reported that the MRP had high DPPH scavenging and antimicrobial activity (46), and we speculated that MRPB may also have some bioactivities, and thus contribute to the decrease of ALAT.

Increased absolute weights of the kidneys in the female group fed with 9% MRPB was also observed in the present study. However, there was no significant difference in relative weights among the three treatment groups fed different amount of MRPB compared with those of the control group. And no treatment-related histopathological changes in the organs of rats receiving MRPB were observed, either. On the other hand, though a slight increase of urea in rats received high dose of MRPB was noticed, there was no statistical difference in both male and female SD rats. These results suggested MRPB had no toxicity on kidneys.

Previous animal studies showed that the dietary intake of polycyclic aromatic hydrocarbons (PAHs) could increase the levels of tumors, due to the formation of HAs during the heat-induced Maillard reaction (47). However, no inflammation was observed in lungs, and nor were simple cysts of the ovaries or other adverse symptoms observed in both male and female rats fed with selected dose of MRPB in this study. In summary, the dietary administration of MRPB for 13 weeks in SD rats was found to have no significant toxic effect both on male and female rats at 1, 3, and 9% MRPB.
